# The role of visibility as a predictor of children’s location choices in outdoor school grounds across age and gender

**DOI:** 10.3389/fpsyg.2026.1832795

**Published:** 2026-05-13

**Authors:** Mine Sak-Acur, Kerstin Sailer

**Affiliations:** The Bartlett School of Architecture, University College London, London, United Kingdom

**Keywords:** affordances, ball games, gender differences, heatmaps, school playgrounds, space syntax, visibility, visual depth

## Abstract

Children’s activities in school grounds are shaped by spatial design, affordances and social dynamics rather than preference alone. This study examines how children of different ages and gender use playground spaces, and how the design and visual depth of the environment shape these dynamics. Playground observations were conducted in two primary schools, one with integrated ball game fields and one with spatially separated fields. A novel method based on ten-second movement trajectories was developed to capture the fluid nature of playground life, from which heatmaps of use were produced. Playground use was analyzed using descriptive statistics and mixed-effects regression models incorporating Visual Mean Depth (VMD), a metric describing visual centrality. VMD significantly predicted children’s location choices, with associations varying by age and gender. Integrated ball game layouts in School A increased boys’ dominance over central and visible areas, pushing anyone not engaged in ball games toward the periphery. By contrast, School B’s separation of sports fields supported more equitable use of central areas, enabling younger children and girls to share visible and non-visible spaces. These findings highlight visibility as a key dimension shaping spatial use, showing that while age and gender matter, design choices, such as the placement of ball game fields, amplify or mitigate these patterns. In this sense, the visibility properties of playground environments shape the affordance landscape within which different forms of play become possible for different age and gender groups. The study underscores the need to consider not only equipment variety but also spatial configuration in playground design to support more equitable use of outdoor spaces of primary schools.

## 1 Introduction

Outdoor school grounds are crucial in children’s lives, as children spend most of their day at school. During the school day, time spent outdoors during breaktimes is among the few meaningful opportunities when children can choose their own activities, interact and communicate with peers on their own terms, release energy and play (Baines and Blatchford, 2010; Pellegrini, 2005). However, children’s activities and play are neither solely dependent on personal preferences nor random; rather, they are significantly influenced by spatial design and the affordances of the environment.

Affordances are a useful concept in understanding human-environment relationship, as it concerns how environments provide possibilities for action for those who perceive and use them (Gibson, 1979; Heft, 2001). According to Gibson (1979), an environment provides information that organisms can pick up through perception and can only be acted upon if the information is available to perception. A person’s visual access is therefore a deciding factor for which parts of the environment are perceptually available and can be detected and acted upon. Movement becomes important in this sense, as different parts of the environment become visible (i.e., visually accessible) and reveal new possibilities for action.

From a first-person perspective, visual access provides information about where one can go, who may be encountered, and what actions appear possible from a given position. Gibson (1979), Ittelson (1973), as well as Appleton’s (1975) prospect–refuge theory all emphasized the role of visually accessible fields, or vistas, in shaping how people cognitively and behaviorally engage with their surroundings (Montello, 2007). Visual access therefore structures the perceptual field within which affordances are detected and acted upon, as the range of visible locations shapes expectations about potential actions and encounters.

Beyond shaping the perception of space, visual access also influences the social conditions of interaction. As Peponis (2024, p. 28) notes, visual access creates a “field of co-presence” in which seeing is inextricably tied to the possibility of being seen. Behaviors within more visible and less visible areas might create differences, as people tend to present themselves differently depending on the presence of others (Goffman, 1971). Through these visual relations, spatial configurations organize patterns of exposure, concealment, encounter, and awareness of others. Visual access therefore does not simply support orientation or navigation; it also shapes opportunities for watching, waiting, approaching, joining, or avoiding others.

Visually accessible areas, and therefore fields of co-presence, are inevitably related to how the environment is configured: where the gaps, walls, solids or transparencies are located. Through an “architecturally-motivated extension of Gibson’s theory” Benedikt (2022, p. 244) represented a person’s visual access with an isovist, which refers to the area visible from a single location, or an individual’s visual field or vista (Benedikt, 1979). Building on this understanding, the space syntax field extended this concept and introduced visibility-based spatial analysis to examine how spatial configuration influences perception, movement and behavior (Turner et al., 2001). Rather than focusing solely on geometric layouts or distances, these approaches model patterns of intervisible locations to analyze how spatial structure shapes movement and behavior. Previous research using space syntax methods across different settings, for example, Zook and Sailer (2022) in hospitals, Sailer and Koutsolampros (2021) in offices, and Tzortzi and Fatah (2024) in museums, demonstrated that what can be seen from a given location strongly influences how buildings are understood, experienced, and used. There are also applications to urban parks (Ascensão et al., 2018; Zhai and Baran, 2013) and public gardens (Li, 2011) which show the merit of visibility based analysis in landscapes and urban public areas that are conceptually similar to playgrounds as bounded open spaces.

By describing how visibility is structured across space, these analytical approaches make it possible to examine how environments organize the affordances available to users. Locations that are visually open, central, or highly connected may afford different opportunities for participation, observation, or interaction than more visually segregated areas (Peponis and Wineman, 2002). In this sense, visibility contributes to shaping the affordances within an environment by influencing which possibilities for action may become perceptually available from different locations.

However, affordances are not perceived uniformly. Although many possibilities for action may be present, individuals tend to respond to those that stand out as most relevant in a given moment (Rietveld and Kiverstein, 2014). Some affordances exert a strong motivational pull, sometimes described as “soliciting” action (Siegel, 2014). The sensitivity to pertinent affordances is formed by an individual’s goals, concerns, and past experiences, such that one affordance that offers the strongest solicitation does not immediately captivate us; we are primarily responsive to those that are most relevant in a given moment rather than the entire constellation of affordances (Ridderinkhof et al., 2011). Furthermore, this process is also socially mediated. What one individual perceives as inviting or demanding action may not even register for another, depending on gendered expectations, cultural scripts, or habitual roles (McClelland and Sliwa, 2023). Therefore, each child’s affordance landscape, which could be defined as their overall perceptual field of possible actions, is shaped by developmental stage, prior experiences, skills, social expectations or institutional norms (Rietveld and Kiverstein, 2014). On the playground, this means that features such as ball game fields, climbing frames, swings, or open lawns may present very different affordance landscapes to children of different ages or gender. For some, a football pitch may afford visibility, competition, and belonging; for others, it may afford risk of ridicule. Likewise, a climbing frame can afford vigorous physical games for one child and imaginative role-play for another. These differences suggest that the same playground environment may present distinct affordance landscapes to different groups of children.

In terms of differentiated use of playground spaces, research has consistently demonstrated that boys and girls use playgrounds in different ways. Boys were more often observed to be engaged in vigorous activities involving team sports and competitive ball games in large groups (Blatchford et al., 2003; Brusseau et al., 2011; Ridgers et al., 2010; Woods et al., 2012). Girls, by contrast, were more frequently observed in conversational activities and sedentary or vestibular forms of play, including jumping rope, gymnastics, climbing, and swinging in smaller, more intimate groups (Blatchford et al., 2003; Lemberg et al., 2023; Pawlowski et al., 2015; Raney et al., 2019).

Age compounds these gender patterns. In the early primary years (ages 4–7 in the UK) boys and girls exhibit similar activity levels, however, by mid-primary years (ages 7–9) a statistically significant gender gap starts to emerge in the literature. While younger primary school children are more likely to engage in mixed-gender play, often in smaller-scale, older children were more often found in same-gender groups, with boys gravitating to large-scale ball games (Lewis and Phillipsen, 1998). Studies show that as they age, girls’ activity levels drop thus creating a gender gap in overall physical activity participation compared with boys (Ariz et al., 2022; Barenie et al., 2024). Swings, jungle gyms, and bars remain popular with girls whilst boys tend to dominate large open areas and courts where competitive games prevail as they get older.

These developmental patterns also manifest spatially. Boyle et al. (2003) found that 9–10 year old boys dominated central areas with team sports like basketball, football, or kickball while girls’ activities were more scattered throughout the playground and included dance routines, walking and talking, jump rope, and creative projects. Importantly, they observed a wide variance among girls, with some comfortable participating in sports and others preferring relational or artistic play. Snow et al. (2019) investigated girls’ perspectives of an ideal playground and found that they differed from sports-oriented affordances, reporting preferences for softer surfaces, a variety of playground equipment and loose parts that could be imaginatively used. Clark and Paechter (2007) further demonstrated how football is viewed as an inherently masculine activity, with boys claiming ownership of pitches and girls frequently needing to negotiate permission to participate, sometimes facing outright exclusion from pitches.

Although the existing literature provides a strong basis for understanding how primary school boys and girls use playgrounds differently across age groups, to our knowledge no studies have investigated these differences in relation to visibility properties (e.g., visually central or visually segregated areas) of playground spaces. Because affordances are perceived selectively and in relation to the actor’s goals, skills, and social positioning, the same visibility conditions may support different forms of activity for different groups of users. There are two recent studies that investigated the affordances of informal learning spaces in secondary schools with regard to their visibility (Fouad and Sailer, 2022); and temporal and spatial limitations on potential affordances in schoolyards created by the visual positioning of teachers and CCTVs (Anteet, 2022), yet neither addressed age or gender differences in playgrounds. Psathiti and Sailer (2023) investigated the visibility of secondary school classrooms and gender, yet their focus and age group differ substantially from the context of primary school play. Kasalı and Doğan (2010) examined age and gender differences in spatial preferences during breaktimes across three different primary schools, but without reference to the visibility properties of space. Notably, they concluded by suggesting space syntax analysis as an angle for future research.

Taken together, previous studies have examined gender differences in playground activity and, separately, the role of visibility in shaping spatial behavior. However, these strands of research have rarely been integrated. As a result, it remains unclear how the visibility properties of playground environments may shape the affordance landscape within which different forms of play become possible for different age and gender groups. Bridging this gap offers an opportunity to understand not only where children play but also how the spatial design of the environment contributes to the organization of play, participation, and social interaction in school playgrounds. To address this gap, this research asks how children use playground spaces by year group and gender, and to what extent the visual depth of spatial configuration influences their use of playgrounds.

## 2 Methods

### 2.1 Case studies: school A and school B

School A and School B are primary schools located in London. They were selected as case studies because they had similarly sized but differently designed outdoor spaces. These outdoor school grounds, which are commonly referred to as playgrounds in the UK, were used by all pupils during breaktimes. At both schools, staying inside the school building during breaktimes was not permitted, except for special circumstances. To prevent the playground from becoming overcrowded, school management at both schools created a staggered breaktime schedule for different year groups. According to this schedule, Years 3 and 4 (ages 8–9) have their breaktime together and once they return to their classrooms, Years 5 and 6 (ages 10–11) begin their breaktime.

**Figure 1:**
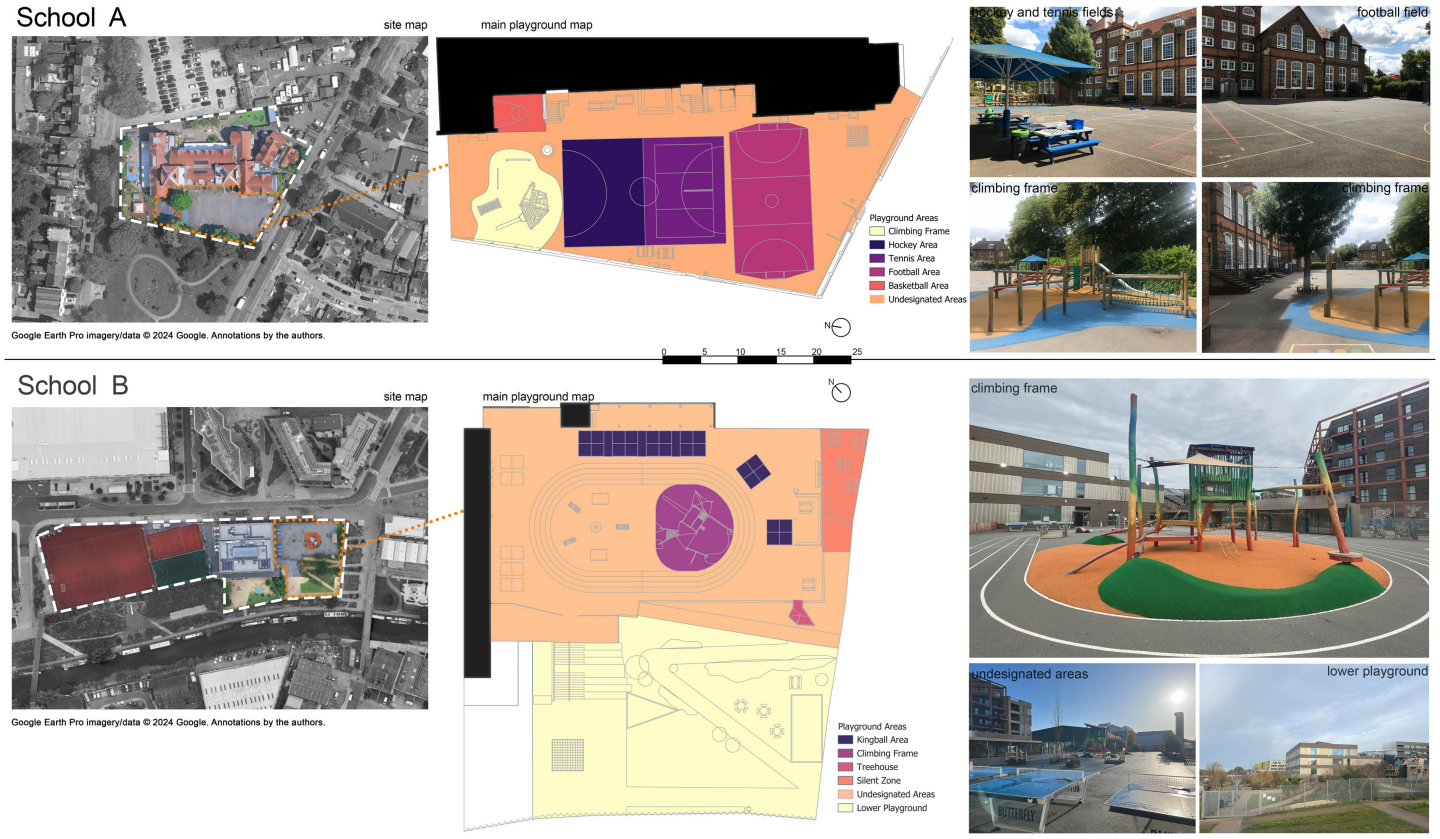
Playground areas of School A (top) and School B (bottom). Google Earth Pro imagery/data © 2024 Google. Annotations by the authors.

School A has a single playground where ball games and other activities coexist. The playground was divided into five zones by the school based on demarcated lines on the floor. The zoning mechanism was designed to prevent conflicts over ball game areas. If children wished to participate in ball games, they had to adhere to the playground rota and were only permitted to play on their allocated days. Children who did not wish to join the ball games were allowed to move freely between zones, provided they did not disrupt the games, and could thus use any remaining spaces outside the designated areas. In this paper, these remaining spaces are referred to as undesignated areas and are defined as playground spaces without a formally assigned play function. Figure 1 shows this functional programming as designated areas (basketball, football, hockey, tennis, climbing frame) and undesignated areas in the playground.

School B has separate football and basketball fields located at the back side of the school building, and the breaktime rota only applied to which group was able to access the basketball and football fields at given times. Therefore, the main playground area did not have any managerial restrictions in terms of general use. However, there was some micro-management in certain areas, such as the treehouse, where only six small and five big children were allowed inside at a time; or the lower playground, which was only open when enough supervising staff were present. The silent zone was also expected to be used for more sedentary activities. Thus, despite the absence of formal boundaries, School B also had certain designated and undesignated areas. Based on observations and interviews with school staff, it was divided into five designated areas (kingball, climbing frame, treehouse, silent zone, lower playground), with the rest labelled as undesignated, similar to School A. A detailed comparison of schools is given in Table 1.

**Table 1:** Comparison of the main characteristics of School A and School B playgrounds.

Characteristic	School A	School B
Location	Primary school in London	Primary school in London
Year groups observed	Years 3–6	Years 3–6
Breaktime arrangement	Staggered; Years 3–4 and Years 5–6 used the playground at separate times	Staggered; Years 3–4 and Years 5–6 used the playground at separate times
Playground size	~1,400 m^2^	~1,600 m^2^
Overall layout	Single main playground	Main playground plus separate football and basketball fields at the back of the school
Ball game fields	Integrated within the main playground	Spatially separated from the main playground
Ball game management	Strict rota for designated ball-game zones	Rota mainly applied to access to football and basketball fields
Formal zoning	Five school-defined zones were established within the playground to support the rota system	Five named playground areas existed with expectations regarding use and behavior, but without the same formal rota-based zoning
Designated areas	Basketball, football, hockey, tennis, climbing frame	Kingball, climbing frame, treehouse, silent zone, lower playground
Undesignated areas	Remaining spaces outside designated zones	Remaining spaces outside designated zones

### 2.2 Playground observations

Common playground observation tools such as Behavior Mapping (Cosco et al., 2010) or the Tool for Observing Play Outdoors (TOPO; Loebach and Cox, 2020) instruct static location markings of children. However, children in a playground are almost impossible to catch sedentary, and even if recorded as static points, it would not have reflected the accurate life within the playground. Therefore, in this study, instead of conventional snapshots, a new method of capturing was developed where the children and their trajectories were recorded over a period of 10 seconds of movement. In both schools, the playground was divided into observable zones and in each zone, following the time sampling protocol of the System for Observing Children’s Activity and Relationships During Play (SOCARP; Ridgers et al., 2010), a random child was selected for observation. The child was observed for 10 seconds following Blatchford et al.’s (1987) rationale that longer observation periods increase the likelihood of a child changing their behavior. They considered 10-second window ideal as it is long enough to capture sufficient information to interpret the behavior, yet short enough to minimize the number of coding decisions required if the behavior changed during the interval. The process of observations was as follows:

- In each zone, a random child was selected for observation.
- The child was observed for 10 seconds.
- The observed child’s movement trajectory was drawn on a playground map.
- The observed child’s year group, apparent gender, activity, and if playing, play type was recorded.
- Moved on to the next random child, and this was repeated until the end of breaktime.

Ten days were spent in each playground recording both in the morning and the lunch break over a period of 3 months in each school. Activity and play categories were developed based on TOPO by Loebach and Cox (2020) and the systematic observation schedule by Blatchford et al. (2003). Detailed descriptions of all items are provided in Supplementary Table S1.

### 2.3 Data analysis

#### 2.3.1 Heatmaps

Manually recorded playground observation data and movement trajectories of all children were digitized using QGIS 3.34 (QGIS Development Team, 2023). After digitizing, a 70 cm buffer was created around each movement trajectory line to represent the area a child takes when moving. The playground maps were divided into 45 × 45 cm cells to account for the average size of a static child, and the number of times each cell was frequented by different categories of children’s trajectories was counted. The frequency of passing each cell was visualized to provide a ‘heatmap of usage’ in the playground.

#### 2.3.2 Visual mean depth

To test whether boys and girls, or younger and older students, have preferences for visually central or peripheral areas, Visual Mean Depth (VMD) was calculated using depthmapX software (depthmapX development team, 2020). VMD represents the average visual “depth” of a location in a spatial system. It is a way of measuring how visually central or segregated a location is within the overall visibility structure of a given space. First, using a grid, the space was divided into cells (we used the same 45 × 45 cm grid to be able to use the same unit of analysis). Two cells were connected if they can see each other without anything blocking the view. By repeating this process for all possible origins and destinations, a spatial network of connections between all the cells was created (Turner et al., 2001).

**Figure 2:**
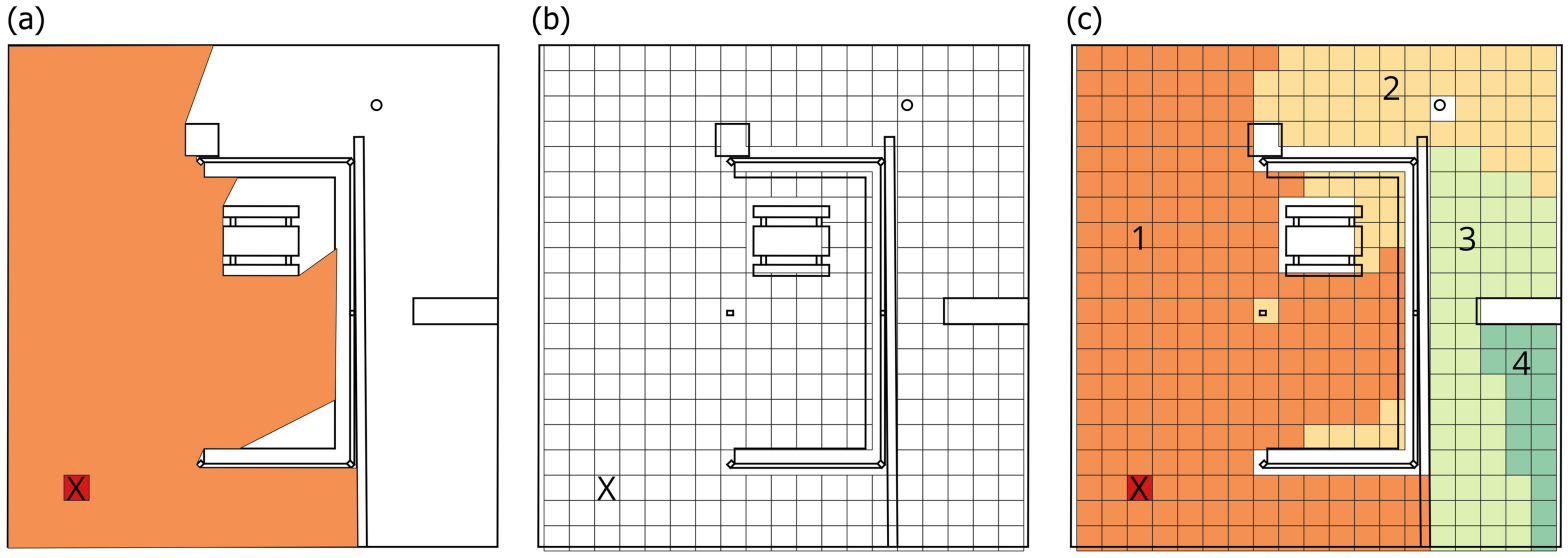
**(a)** Directly visible locations from point X, **(b)** 45 × 45 cm divided map, **(c)** Number of turns needed to see everywhere from point X.

Using this network, VMD is calculated by counting and averaging the number of edges in the shortest visual paths required to connect one cell to all others. Each edge in the system is considered as a ‘visual step’: one step means two cells are directly connected (i.e., visible to each other), two steps mean that to see from one cell to another, there is one intermediate cell in between, and so on (Koutsolampros et al., 2019). Each visual step can be considered as turning around a corner to see somewhere new (Figure 2). Higher VMD values signal that a location is visually deeper (i.e., more strategically hidden) in the system, since the average number of visual turns needed are high. Conversely, lower values signal visually more central locations, which are in closer visual reach to the rest of the spatial system. Essentially, this visibility analysis produces a numerical result coming directly out of the spatial configuration and thus the design of the space.

The playground map to conduct this analysis was drawn based on an average primary school age child’s eye level. The visual obstructions were determined at the level of 120 cm from the ground.

#### 2.3.3 Negative binomial mixed-effects regression analysis

The nature of the collected data was count data (movement trajectory frequencies moving over each cell) and they were not normally distributed, hence linear models were not fit for purpose for this dataset (Hilbe, 2011). After checking overdispersion and zero inflation in the dataset, Negative Binomial Regression analysis was deemed fit.

Fixed effects in the statistical models test the systematic relationships between the predictors and the outcome variables, while random effects account for the variation in the data due to grouping or clustering structures, that are not specifically tested but potentially influence the results (Snijders and Bosker, 2012). In this study, different playground zones might have naturally different baseline numbers of children due to the playground rota and the attraction of equipment. For example, a child might be playing tennis because they like tennis, not necessarily because it is visually integrated or segregated. Therefore, the functional programming of playground areas needed to be accounted for in the statistics. By adding a random intercept for each playground area, it was accounted for zone-level variability in baseline counts and ensured that the effect of visual depth was tested beyond the expected differences between functional areas. Negative Binomial (NB) generalized linear mixed models (GLMMs) were fitted to examine factors influencing spatial use. The predictor, VMD, was evaluated through a sequence of models:

- M0 Null: An intercept-only model with no predictors and no random effects.
- M1 Fixed Effect: A model that included VMD as a fixed effect, without random effects.
- M2 Random Intercept (Zone): A model including VMD as a fixed effect and a random intercept for playground zone, allowing each zone to have its own baseline level.
- M3 Interaction: A model including VMD and its interaction with a grouping variable (gender x year group), in addition to a random intercept for zone.

All statistical analyses were conducted in RStudio 2024.04.2 (Posit team, 2025).

### 2.4 Ethics statement

Ethical approval for high-risk research was obtained from the (University College London Ethics Committee) Research Ethics Committee in August 2023, before commencing the fieldwork. After the headteacher’s written permission, information sheets and opt-out forms were sent to all parents/carers of years 3, 4, 5 and 6 students. Three students in School A and one student in School B were opted out of the observation study by their parents, thus they were not observed.

## 3 Results

### 3.1 Participant characteristics and overall activity patterns

To establish the baseline patterns of playground activity, we begin by describing the characteristics of the observed participants and the overall distribution of activities across two playgrounds. A total of 1,245 children were recorded in School A and 1,640 in School B over 10 days of data collection in each. As Table 2 shows, in both schools, the proportion of recorded boys and girls was almost equal (School A: 50.8% vs. 49.2%; School B: 49.1% vs. 50.9%), and the distribution between younger (Y3/4) and older (Y5/6) cohorts was similarly balanced (School A: 51.1% vs. 48.9%; School B: 51.3% vs. 48.7%). In School A, 82.7% of observations were recorded as play, compared to 72.5% in School B, meaning that play dominated breaktime activities in both cases accounting for approximately three-quarters of all recorded behavior. Conversations accounted for 9.6% of observations in School A and 20.6% in School B, reflecting a greater tendency for chatting without a play element in School B’s playground. Inactivity remained rare across all groups. These patterns establish play as the primary mode of playground engagement.

**Table 2:** Descriptive statistics of observed children by gender, year group, and activity type in School A and School B.

Category		School A			School B		
		Freq.	%	Total	Freq.	%	Total
Gender	Male	633	50.8%	1,245	805	49.1%	1,640
	Female	612	49.2%		835	50.9%	
Year group	Year 3 and 4	636	51.1%	1,245	841	51.3%	1,640
	Year 5 and 6	609	48.9%		799	48.7%	
Activity type	Other	96	7.7%	1,245	113	6.9%	1,640
	Just conversation	119	9.6%		338	20.6%	
	Play	1,030	82.7%		1,189	72.5%	

Furthermore, Table 3 shows the number of children recorded in each area. In both schools, the largest group of children was recorded in undesignated areas (A: 33%; B: 43%). In School A, these areas contained loose play materials, such as skipping ropes, wooden blocks, hula-hoops, coloring books and pencils. Children also played with sticks, rocks and soil they found under the hedges surrounding the playground. They engaged with these natural and manufactured loose parts, but also undesignated areas were ideal for their chasing-catching games as they could play there without disturbing the ongoing ball games. On the other hand, School B provided loose parts only in the lower playground area, which was only accessible when the school had enough supervising staff. As a result, children were unable to play regularly with many of the loose elements. The undesignated areas in School B had fixed materials such as benches, ping-pong tables and wooden bridges. Therefore, the most commonly observed behaviors in undesignated areas of School B were sitting and chatting on benches, playing ping-pong, chasing catching games and reading.

**Table 3:** Playground areas and observation frequencies in both schools.

School A				School B			
Playground areas	Freq.	%	Total	Playground areas	Freq.	%	Total
Basketball area	35	2.8%	1,245	Kingball area	178	10.8%	1,640
Climbing frame	289	23.2%		Climbing frame	460	28.0%	
Football area	164	13.2%		Lower playground	102	6.2%	
Hockey area	193	15.5%		Silent zone	109	6.6%	
Tennis area	152	12.2%		Treehouse	79	4.8%	
Undesignated areas	412	33.1%		Undesignated areas	712	43.4%	

The second largest group of observed children was on climbing frames (A: 23%; B: 28%). Similar to undesignated areas, children engaged in a variety of activities on and around climbing frames, from playing organized games to chatting with friends. This was followed by ball game zones with hockey (15%), football (13%), tennis (12%) and basketball (3%) areas in School A. In School B, the next most frequently used areas were the kingball area (11%), silent zone (7%), lower playground (6%) and treehouse (5%). In the silent zone and treehouse, children were mainly observed in imaginative and restorative play, while in the lower playground they were mostly engaged with loose play elements alongside imaginative and physically active play.

### 3.2 Gender and age differences in activity and play

**Figure 3:**
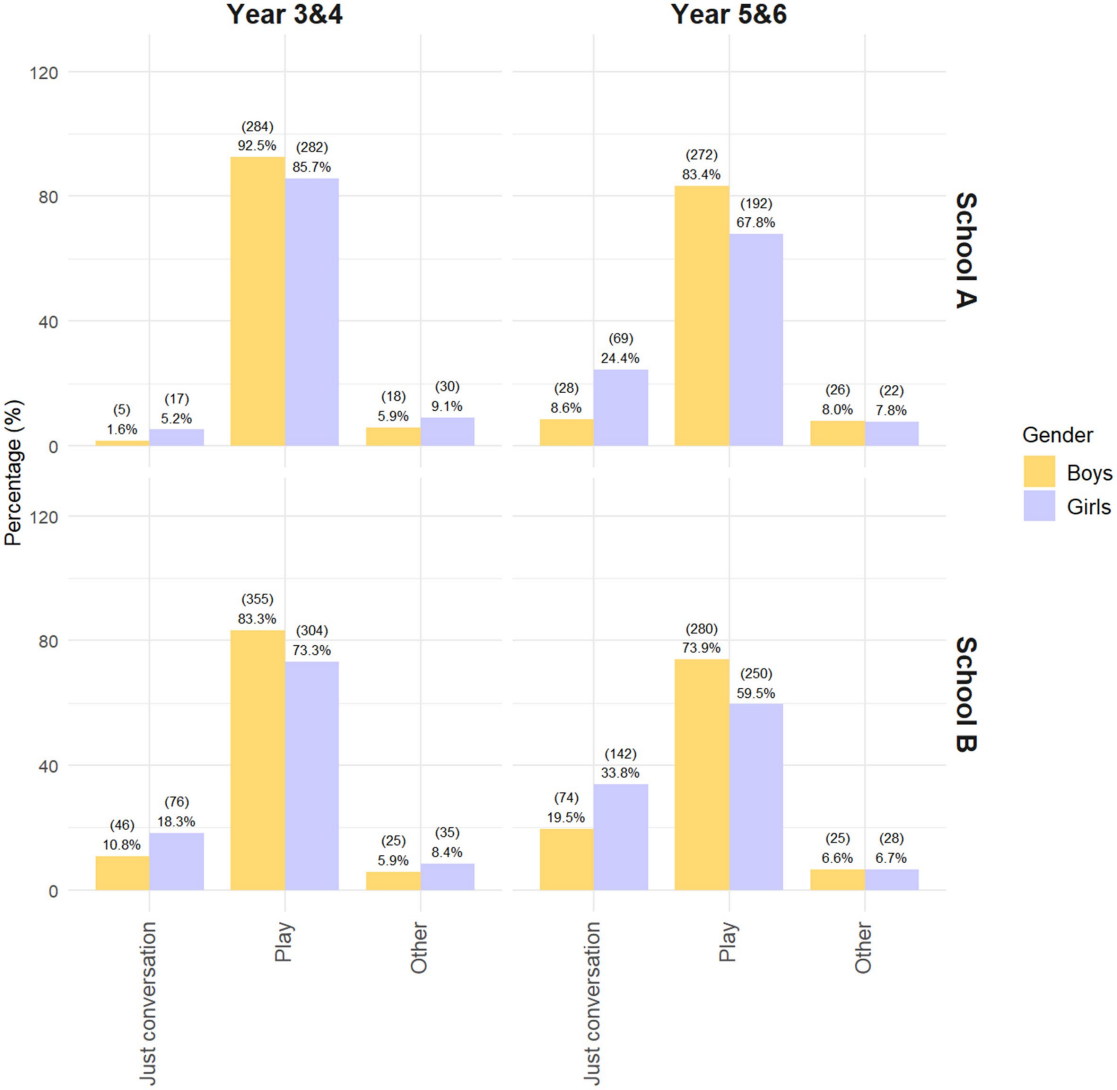
Distribution of activity types by gender and year group in School A and School B.

Having established the overall activity patterns, we next examine whether playground engagement varies by gender and year group. In both schools, there were clear differences in activity engagement across year groups and gender as can be seen in Figure 3. Play declined with age, most notably among girls, while conversational activities increased. In School A, play engagement among girls dropped from 85.7% in Years 3 and 4 (Y3/4) to 67.8% in Years 5 and 6 (Y5/6); in School B, the corresponding decline was from 73.3 to 59.5%. Older girls showed the highest proportion of conversational activity in both contexts. Activity type differed significantly by year group and gender in both schools (School A: *χ*^2^(6) = 105.95, *p* < 0.001, Cramér’s *V* = 0.21; School B: *χ*^2^(6) = 75.62, *p* < 0.001, Cramér’s *V* = 0.15). In both contexts, standardized residuals indicated that older girls (Y5/6) were overrepresented in conversational activities and underrepresented in play. Younger boys (Y3/4) were underrepresented in conversations in both schools and overrepresented in play, particularly in School B (full results in Supplementary Tables S2, S3).

**Figure 4:**
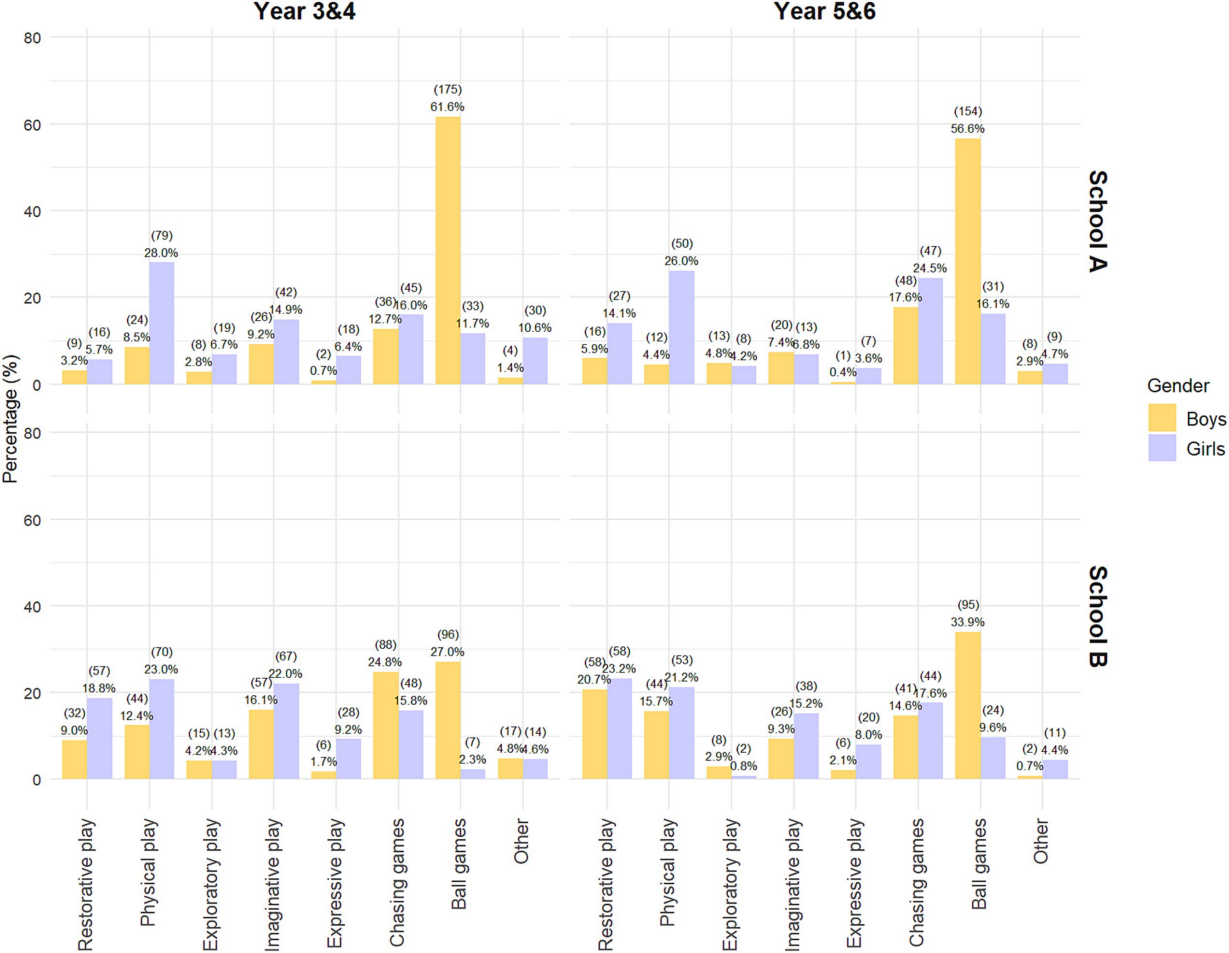
Distribution of play types by gender and year group in School A and School B.

As Figure 4 illustrates, play type distributions differed significantly by year group and gender in both schools (School A: *χ*^2^(21) = 311.25, *p* < 0.001, Cramér’s *V* = 0.32; School B: *χ*^2^(21) = 206.46, *p* < 0.001, Cramér’s *V* = 0.24). In both contexts, standardized residuals showed that ball games were strongly overrepresented among boys across year groups and underrepresented among girls. In contrast, girls, particularly Y3/4, were more engaged in a wider variety of play types, including physical, imaginative and expressive play, all showing strong positive deviations in both schools. Overall, ball games emerged as the primary driver of gendered differences in play, with other play types contributing comparatively smaller deviations. These findings indicate that age and gender differences in playground behavior are not limited to whether children play but also to how play is organized and which forms of play take place.

### 3.3 Heatmaps of spatialized behavior

**Figure 5:**
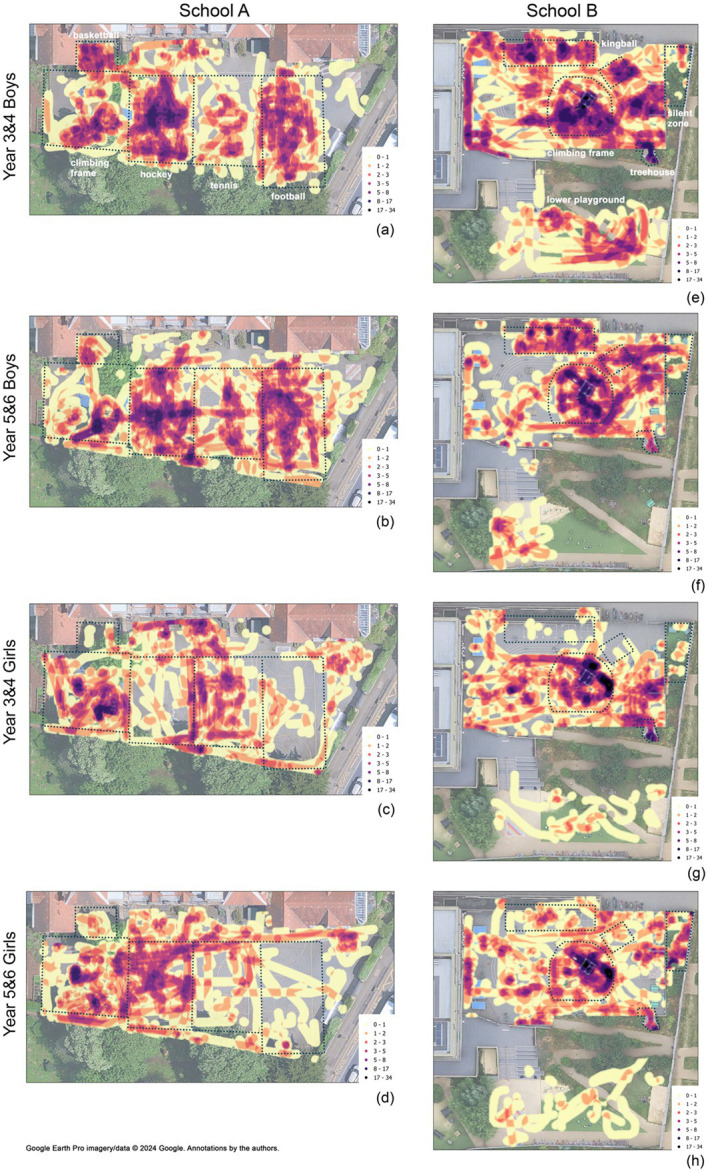
School A: **(a)** Y3/4 Boys, **(b)** Y5/6 Boys, **(c)** Y3/4 Girls, **(d)** Y5/6 Girls; School B: **(e)** Y3/4 Boys, **(f)** Y5/6 Boys, **(g)** Y3/4 Girls, **(h)** Y5/6 Girls. Google Earth Pro imagery/data © 2024 Google. Annotations by the authors.

To explore how these behavioral differences are reflected spatially, we mapped children’s movements across the playgrounds using heatmaps. The heatmaps of use by different groups of children visually reinforced the statistical differences identified in play preferences between groups, as highlighted in Figure 5. They illustrate that these differences are not limited to the types of play they engage in but are also reflected in the spatial patterns of their movement across the playground. In other words, variations in play types are closely linked to how boys and girls of different year groups occupy different areas of the playground. Boys’ movement visibly aligns with the ball game areas in both playgrounds. In School A, vertical up-and-down movement between the two goalposts of football are particularly clear in the boys’ map, showing that the movement patterns are affected by the game played. Additionally, the basketball area in the upper left corner, the hockey field and the two sides of the tennis net are particularly used. They illustrate boys’ dominant use of central playground spaces, in contrast to the smaller, more peripheral groupings observed among girls. In School B, boys primarily occupied the kingball areas (also known as king square or four square), which was the only permitted ball game in the School B’s main playground as children needed to go to the separate sports fields to play basketball and football. The climbing frame areas were a frequent destination for boys in both schools.

The movement patterns of girls differ across schools. While more scattered in School A, the hotspots are more condensed at certain locations in School B. In School A, girls’ movement did not take up space in the center of the zones but instead tended to follow the edges of the designated areas and the lower edge of the playground. It appears that girls moved around the zones, avoiding the ball games happening in the center, and navigated their way along the zone borders. While there is very little movement in the football area, a small area of higher frequency is visible in the upper right corner of the playground, where children were mostly engaged in skipping. In School B, girls’ movements formed one major hotspot at the monkey bars and climbing frame tower, alongside several smaller clusters across the playground. Compared to boys, they occupied the Silent Zone more frequently. As in School A, movement near ball game areas, here the kingball areas, was minimal for girls.

Across genders, the high density of movement around the climbing frame and monkey bars was consistent in both maps. They highlight the climbing frame as one of the most densely used areas for all students, while the monkey bars stand out as especially popular among girls which confirms the high numbers of vestibular play instances.

### 3.4 Visibility and spatial configuration effects

So far, we have shown that children’s play type choices and location aggregation on playgrounds differ by gender and year group. To test whether children’s location choices are influenced by the visibility relationships beyond the equipment provided in different areas, we ran a Negative Binomial Regression analysis where VMD was the predictor, and the number of children was the outcome variable. This way, we aimed to test whether the visual depth of the spatial system arising from the design of the playground (i.e., allocation and configuration of equipment and the overall layout) affected the preferences of different groups. As explained in the methodology, we built four models and compared them based on deviance, AIC, BIC, and statistical significance of predictors. The best-fitting model (M3), which included gender × year group interactions, significantly improved model fit over simpler models (M0, M1, M2) in both schools (likelihood ratio tests, *p* < 0.001). Thus, M3 was selected as the appropriate model for analysis.

Focusing on the best fitting model M3, the interaction slopes were estimated separately for each group and converted into interpretable percentage changes in counts by calculating Incident Rate Ratios (Piza, 2012). This transformation allowed direct comparison of how a one-unit increase in VMD is associated with the relative change in children’s presence across gender and year group categories. To ensure the significance is assessed at the correct level, the *p*-values calculated to correspond to the group specific slopes rather than the overall model.

**Figure 6:**
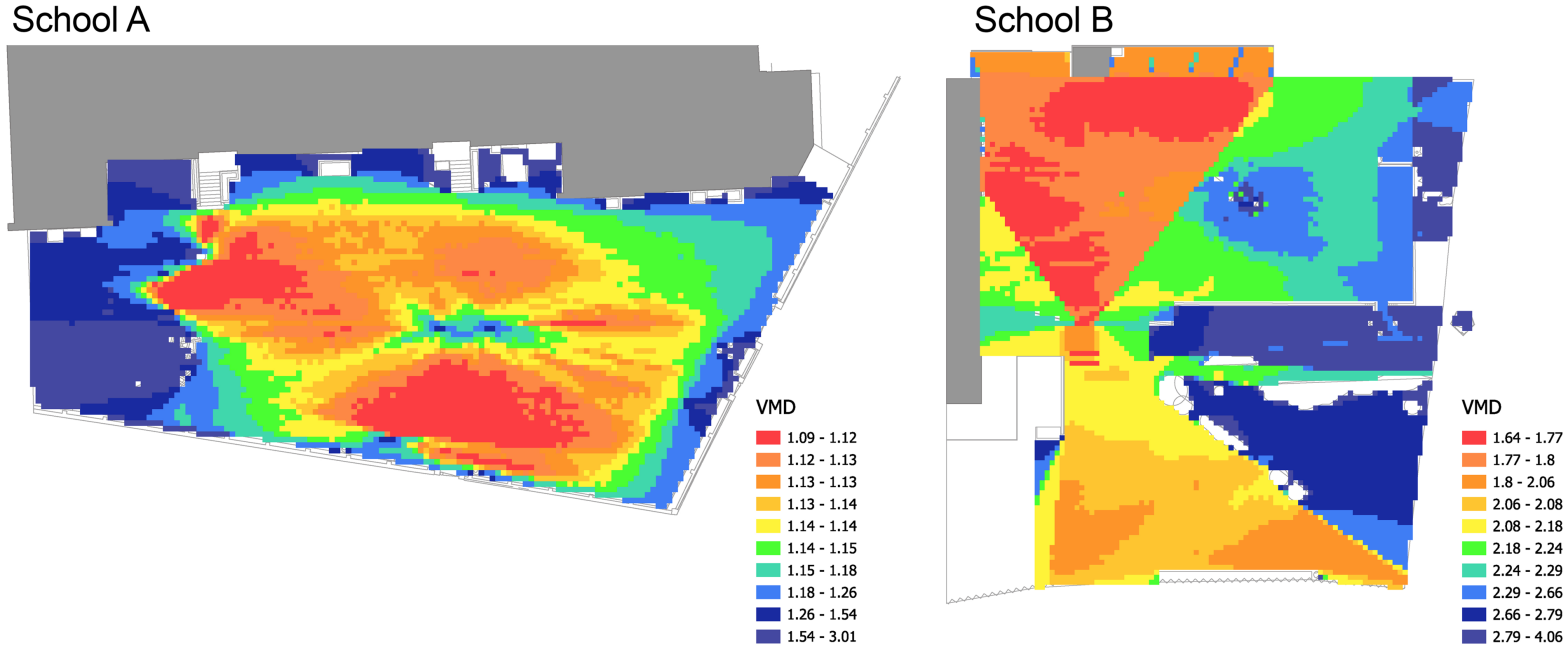
Visual mean depth (VMD) results for School A (left) and School B (right) playgrounds.

The range of VMD values fell between 1.09 and 3.0 in School A with an average of 1.22 and between 1.63 and 4.05 with an average of 2.23 in School B (Figure 6). This indicates that School B had a visually deeper layout on average, meaning that more visual steps were needed to see all parts of the playground.

The effects of VMD in the regression models (Table 4, full results in Supplementary Table S4) were consistently negative across groups and schools, except for Y3/4 girls in School A which showed a 49% increase in counts with one-unit increase in VMD. It stands in contrast to all other groups since higher mean depth, hence higher visual segregation, was associated with lower numbers of children in general, particularly for older boys (−47% in School A, −51% in School B). This suggests that younger girls in School A were more present in the visually deeper locations, whereas other groups preferred more visually integrated locations. Y5/6 girls in both schools showed negative and smaller associations with VMD. A one unit increase in VMD led to a 21% decrease in the number of Y5/6 girls in School A and a 12% decrease in School B, which means their likelihood of being in visually central areas was higher, thus highlighting a potential shift in spatial preferences with age.

**Table 4:** Negative binomial regression results: percentage change in counts per 1-unit increase in VMD.

School	Group	*p*-value	% change
School A	Y3/4 Boys	<0.001***	−24%
	Y3/4 Girls	<0.001***	49%
	Y5/6 Boys	<0.001***	−47%
	Y5/6 Girls	0.001***	−21%
School B	Y3/4 Boys	<0.001***	−36%
	Y3/4 Girls	<0.001***	−30%
	Y5/6 Boys	<0.001***	−51%
	Y5/6 Girls	0.002**	−12%

Signf. codes: <0.001***, <0.01**, <0.05*. Positive = increase in counts; Negative = decrease in counts.

**Figure 7:**
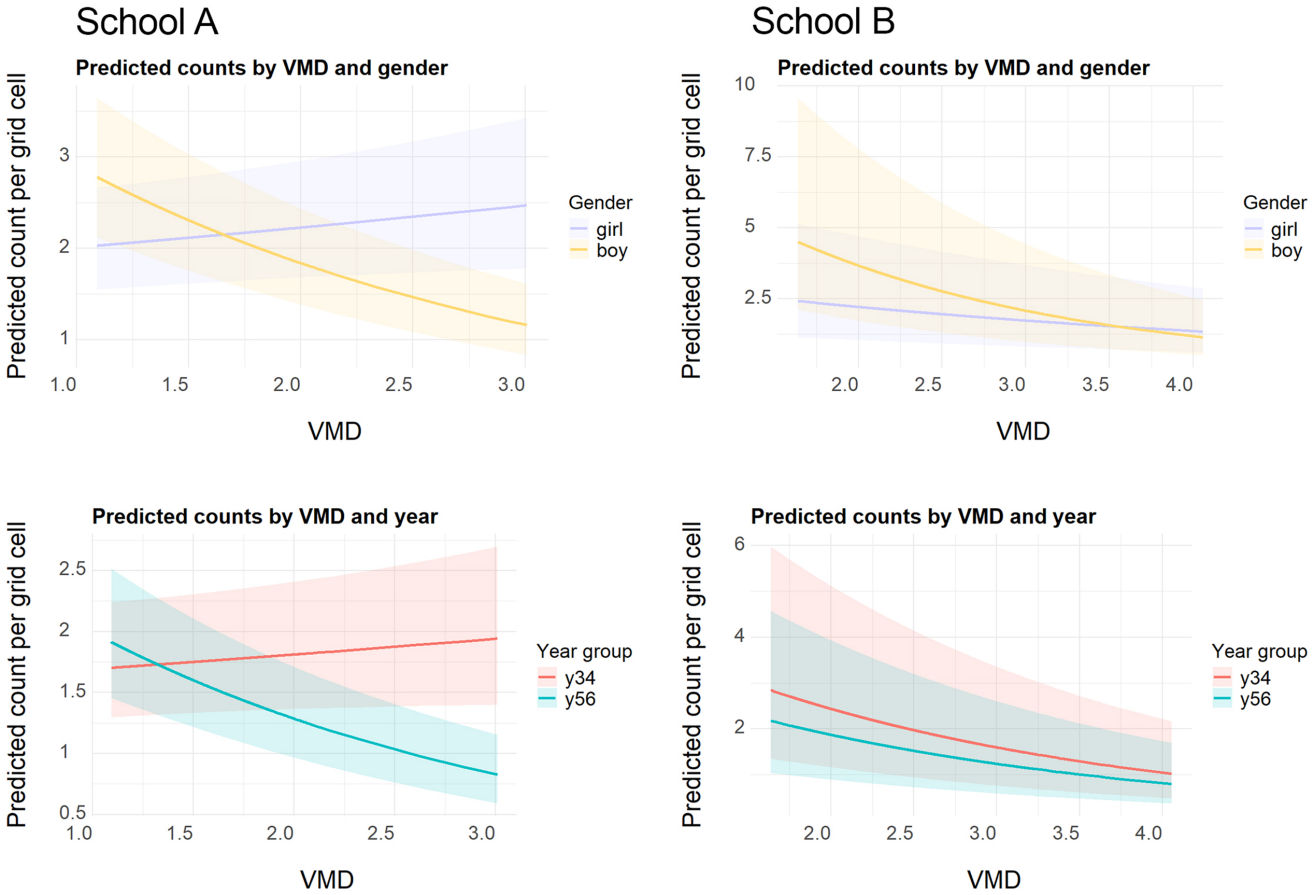
Predicted counts of children by gender (top) and year group (bottom) per grid cell across values of VMD based on the negative binomial model M3. Shaded bands = 95% confidence interval.

Overall, boys consistently preferred visually central areas (lower VMD). Y3/4 girls in School A frequently diverged from other groups, favoring deeper spaces (higher VMD). Older girls tended to align more closely with boys, particularly in their avoidance of deeper spaces. Figure 7 shows gender and age differences visually. School A shows a greater discrepancy between gender and year groups since as the line of one group inclines, the other one declines. In contrast, School B shows lines following the same direction across groups. This difference between schools might be stemming from the fact that sports fields in School A were placed within the main playground area and mostly occupied by boys whereas School B had separate sports fields and the main playground was used similarly across different groups. To test this possible explanation, in the next section the same analysis will be conducted after excluding all observations of ball games.

### 3.5 Excluding ball game observations

In a playground where ball game areas are set by the school management, children who want to play ball games will automatically end up using these allocated zones. This overrules the idea of choosing a place. Therefore, to disentangle configurational influences from institutional allocation, children observed playing ball games were excluded from the subsequent analysis. Since boys were disproportionately engaged in ball games, this adjustment shifted the prior frequencies in the dataset. Table 5 (full results in Supplementary Table S5) shows how the remaining children’s movement patterns varied by gender and year group in relation to VMD across both schools.

**Table 5:** Negative binomial regression results: percentage change in counts per 1-unit increase in VMD with data excluding ball games.

School	Group	*p*-value	% change
School A	Y3/4 Boys	<0.001***	179%
	Y3/4 Girls	0.83	−2%
	Y5/6 Boys	<0.001***	70%
	Y5/6 Girls	<0.001***	−57%
School B	Y3/4 Boys	<0.001***	−23%
	Y3/4 Girls	<0.001***	−32%
	Y5/6 Boys	<0.001***	−28%
	Y5/6 Girls	0.10	−7%

Signf. codes: <0.001***, <0.01**, <0.05*. Positive = increase in counts; Negative = decrease in counts.

**Figure 8:**
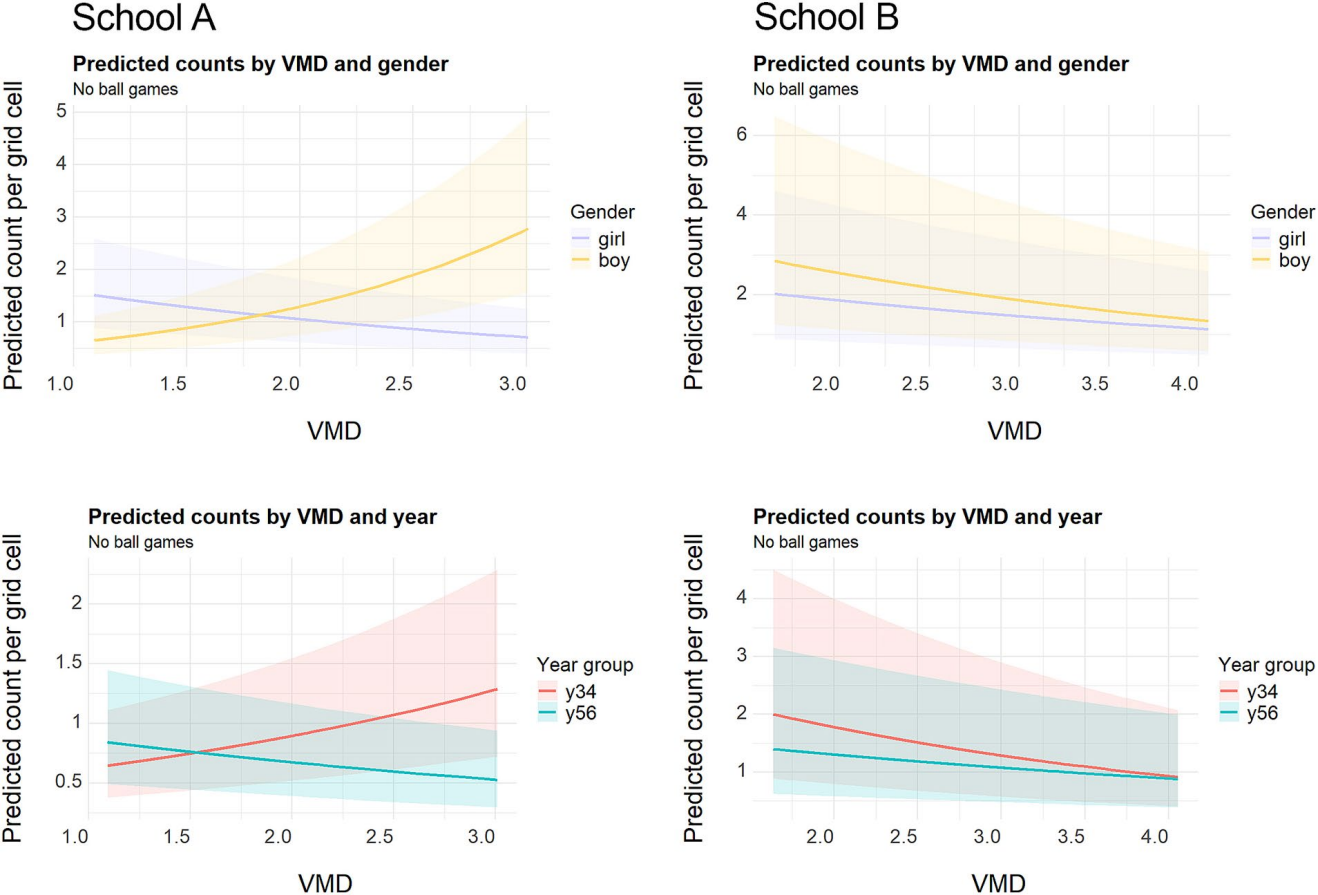
Predicted no ball game counts of children by gender (top) and year group (bottom) per grid cell across values of VMD based on the negative binomial model M3, excluding ball games. Shaded bands = 95% confidence interval.

In School A, boys became strongly associated with visually deeper areas in contrast to the results presented in the previous section. Counts increased by 179% for Y3/4 boys and 70% for Y5/6 boys per unit increase in VMD. By contrast, Y5/6 girls showed a 57% decrease, while Y3/4 girls showed no significant association. School B boys continued to avoid deeper spaces, though the decline was smaller (−23% in Y3/4 and −28% in Y5/6). Girls’ patterns shifted as well: the positive association of Y3/4 girls with deeper areas in School A (+49%) disappeared once ball games were excluded (−2%, non-significant), while in School B the negative association remained (−30% previously vs. –32% now). Together, these results suggest that boys in School A actively used deeper areas when free from ball games, while in School B both genders tended to cluster in more visually central spaces. The marginal effects plots in Figure 8 illustrate this divergence: the upward slopes for boys and Y3/4s in School A stand in contrast to the downward slopes for both genders and year groups in School B.

Overall, excluding ball games uncovered clearer contrasts in how boys and girls from different year groups used playground spaces. Excluding ball games substantially altered the spatial associations, particularly for boys in School A, whose negative relationships with VMD became positive. Essentially, boys not engaged in ball games were mostly found in deeper and less visible locations of the playground. For girls, the distinctive positive VMD association of Y3/4 girls in School A disappeared while older girls’ negative association became stronger. On the contrary in School B, Y5/6 girls’ negative association became non-significant while for Y3/4 girls no substantive changes occurred.

## 4 Discussion

This study examined how playground design and visibility relations shape children’s spatial use of playgrounds and play across age and gender. Across both schools, play declined with age, boys and girls engaged in different play types and occupied space in different ways. By comparing two playgrounds with integrated versus spatially separated ball game fields, the findings showed that integrated and separated ball game fields created different affordances. As ball games are highly space-consuming, their placement had a significant impact on who occupied central and peripheral spaces and, by extension, who was rendered more or less visible. In School A, where ball games were integrated into the main playground, boys’ use of central areas restricted other play forms to the left-over spaces. In contrast, School B’s separate fields allowed a wider range of activities to flourish in the main playground, enabling girls to claim more central grounds.

### 4.1 Age and gender differences in play

In line with the literature, our findings in both schools suggest that play declined with age, especially among girls, while conversations increased. This trajectory reflects broader developmental shifts discussed in the literature, where older children, particularly girls, transition toward more verbal social interactions and away from physically active play (Blatchford et al., 2003; Lemberg et al., 2023; Ridgers et al., 2010). The decline in play and the rise in chatting can be understood as a shift in perceived affordances where older children become more attuned to the potential for verbal interactions than to physical play opportunities.

### 4.2 Spatial preferences by gender

Ball games were strongly associated with boys in both schools while girls engaged in a bigger variety of play types including vestibular, imaginative, and expressive play, as suggested by other studies (Martinez-Andrés et al., 2017; Vaughter et al., 1994). Especially the heatmaps of School A showed boys concentrated in ball game areas, which by design allowed them to occupy central playground spaces. They illustrated how boys’ movement aligned with football and kingball spaces, while girls were more often located at the edges, avoiding these zones. Girls in both schools tended to avoid ball game areas and preferred the monkey bars which are linked to vestibular play. In School A in particular, girls’ movement was more peripheral and scattered whereas School B offered larger ball-free spaces that produced more distributed hotspots. Therefore, in School B, girls were not confined to peripheries as there were not any particularly dominating activity on the central playground.

### 4.3 Playground design and spatial dominance

Results of this study come from two distinct playgrounds with multiple sports fields: one integrated into the main playground (School A) and another separated from it (School B). While Andersen et al. (2019) show that the presence of multiple sports fields is associated with increased activity for both genders, our findings suggest that the spatial placement of these fields within the playground is equally critical. In School A, integrated ball game fields concentrated activity in central areas, whereas in School B, separating these fields allowed a broader range of play types to occupy the main playground.

Integrated ball game fields in School A created a salient affordance for boys, drawing them into central areas. This shows that the preference was not for centrality itself but for ball games which were designed to be central, thereby allocating those areas to whoever played ball games. This is similar to Ndhlovu and Varea’s (2018) findings where the only large open space was occupied by football limiting all other students use of it, and with Martinez-Andrés et al.’s (2017) and Swain’s (2000) findings that those not choosing to play football were confined to the peripheries of the playground. Furthermore, our findings support Raney et al.’s (2023) argument that the playgrounds are not being used to its full potential when a significant portion of its area is devoted to sports fields and ball games. Thus, ‘boys dominating playgrounds’ should actually be read as ‘boys dominating ball game fields’ as it is the responsibility of playground design to decide whether these fields are placed in the middle of the playground or not.

Furthermore, the dominance of ball games in School A not only shaped spatial use but also influenced overall activity patterns. As shown by the results, more than one-third of all play in School A constituted ball games, compared to less than one-fifth in School B. As the two playgrounds were comparable in size, this difference likely occurs because of the design. School A’s ball game fields were integrated within the main playground whereas School B’s were located separately, allowing more space for other play types to flourish in the main playground.

The only exception to the overall pattern of gendered spatial use was the climbing frame and monkey bars. In both playgrounds, these fixed play structures were highlighted in the heatmaps as the most used spaces by all children. Since fixed structures offer differentiated affordances, such as climbing, swinging, jumping, running, hiding and much more, children outside of ball games were able to find something appealing. This resonates with other observational studies showing fixed playground equipment as the most used spaces (Farley et al., 2008), with some highlighting more frequent girls usage compared to boys (Amholt et al., 2022; Pawlowski et al., 2015). These findings illustrate how a single multi-functional element can provide a rich and equally accessible affordance landscape for many children.

### 4.4 Visibility relations shape playground use

In terms of visibility relations, VMD had negative associations with all children in School B, meaning, the deeper the location visually, the less likely it was to encounter a child. However, in School A, the pattern was more complex. When ball game observations were included, the results show that Y3/4 girls were more likely to be in deeper spaces while all others were less likely. When ball game observations were excluded, the significance of Y3/4 girls’ association with deep spaces diminished. This indicates that girls appeared relatively more associated with deeper spaces not because they actively sought them, but because large numbers of boys occupied the shallow, central areas. In other words, the concentration of boys playing ball games in central locations created a contrast, making girls appear to be in deeper locations. Once ball-game observations were removed, Y3/4 girls no longer stood out as being located in deeper spaces. This is an important finding, as it highlights that younger girls do not actively seek deeper spaces but only appear to do so in comparison to boys. This finding complements Aminpour et al.’s (2020) study where they define in-between spaces as small enclosures located at the edges of the grounds, which are mostly visually segregated areas. They show that girls’ frequent use of in-between spaces is often a result of exclusion from centrally located sports courts rather than an active preference for margins, similar to our results.

In School B, where ball games did not dominate the central playground, both younger and older girls were found in visually central (low VMD) areas both before and after the removal of ball game observations. As ball game fields did not take up all the central grounds in School B, children who were not engaged in ball games had the chance to use these areas. This suggests that, in the absence of ball game domination in the central playground, girls were also willing to use these spaces. Combined with the results in School A, where girls did not actively seek deeper areas, this is a novel finding that was not explored in the literature in such detail before and adds an important nuance to the ongoing discussions about ball game domination in school playgrounds.

A shift following the removal of ball games appeared among boys in School A. While boys of all year groups were originally associated with visually central locations, without ball games all became associated with deeper locations. Y3/4 and Y5/6 boys not engaged in ball games were 179 and 70% more likely, respectively, to be found in deeper spaces. Although ball-game-playing boys did not directly alter girls’ associations, they significantly shaped the behavior of other boys. This almost confines boys’ spatial behavior to two primary modes: a ball-game mode (central, visible, low VMD) and a non-ball-game mode (deeper, less visible, high VMD). These findings can also be read alongside Pawlowski et al.’s (2015) identification of gendered recess typologies, particularly the distinction between “soccer boys” and “the nerds” who avoided sports fields and played fantasy games in the shrubby areas of their playground. From a spatial perspective, these typologies can be reinterpreted as practices aligned with different visibility conditions: ball-game play corresponds to visually dominant, central areas, while other activities occur outside these spaces. Our analysis extends Pawlowski et al.’s qualitative insights by showing how such social typologies are actively produced and reinforced through the spatial configuration and visibility structure of the playground.

Importantly, it is shown that visual depth does not replace functional programming as an explanation of playground use, rather it helps explain how the placement of functionally programmed areas within the visibility structure of the playground shapes who uses them and who avoids them. In this sense, visual depth is not only a configurational property but also a social one, as it affects the condition of being more or less visible to others. If visibility is considered a dimension of affordances, children’s location choices may be influenced not only by what an area allows them to do, but also by whether it places them under many eyes or provides relative visual retreat for their selected activities. Returning to Peponis' (2024) concept of fields of co-presence, the visual depth of an environment provides different opportunities for being co-present with others. For example, even if a child enjoys playing football, a football field in the middle of the playground means being directly under many eyes, which some children might avoid not because they do not like football but because they do not want to be observed by many. Thus, the positioning of areas within the playground does not simply distribute activities, it also shapes the social exposure attached to those activities, which possibly influences whether particular spaces are used or avoided by children.

### 4.5 Limitations and future directions

This study has several limitations. The analysis is based on only two case studies within an urban UK context and therefore cannot be generalized beyond them. Still, the study draws on a large and balanced dataset of more than 2,800 children, with near-equal distributions by gender and year group, which strengthens the validity of the comparisons. Future research including a larger and diverse set of playgrounds might reveal different aspects. Additionally, while the spatial analysis provides valuable insights, it cannot fully account for social dynamics such as friendship groups, peer hierarchies, or teacher interventions, all of which profoundly influence playground behavior. By combining the spatial analysis with robust statistical testing that accounted for group interactions and zone differences, we were able to address some of these interacting elements and better understand the relation between space and behavior in the playground. Finally, single-observer research poses some challenges for data collection, including potential observer bias, although it ensures consistency and coherence (Czalczynska-Podolska, 2014). To mitigate this, data collection categories were clearly defined and described beforehand to make no room for interpretation. In cases of indecision, the subject was not recorded.

Despite these limitations, our findings underscore that playground design is not only about the choice of equipment but also about how play areas are configured and positioned. Decisions about the placement of sports fields carry particular importance, because firstly they take up a large amount of space in the playground, and secondly their high demand by boys strongly influences the dynamics of use. Areas for diverse play should not be treated as leftover spaces squeezing children uninterested in ball games into the periphery. Instead, they need to be designed to cater to the diverse needs beyond ball games. Fixed equipment like climbing frames and monkey bars were observed to be used by all children regardless of age or gender, though with different purposes, therefore promising to offer high engagement levels with varied affordances. Playground design needs to consider not only the equipment provided but also the configuration of these elements, as this shapes both movement patterns and the social dynamics of playground life.

## 5 Conclusion

This paper has shown how playground design, the arrangement of spatial elements and visibility relations play a central role in shaping how children experience, access, and participate in playground life. In this sense, this study provides empirical insights into people-environment relations within school playground settings. The main strength of this research lies in adding a spatial dimension to a field that is primarily studied in behavioral and social terms. By considering visual depth as a dimension of affordances and conducting an in-depth spatial analysis of visibility relations, we offer a novel perspective to behavioral data from school playgrounds. The study contributes to environmental psychology by showing how perceptual qualities of the built environment shape the social life of playgrounds, including equitable access to outdoor space and play opportunities. It highlights the value of considering configurational features such as visibility for both design practice and future research.

## Data Availability

The raw data supporting the conclusions of this article will be made available by the authors, without undue reservation.
