# Supplementary material for: The role of visibility as a predictor of children’s location choices in outdoor school grounds across age and gender
Source: Front Psychol. 2026 May 13;17:1832795. doi: 10.3389/fpsyg.2026.1832795 (PMC13212169; doi:10.3389/fpsyg.2026.1832795)
Supplement: Supplementary file 1 [file Table_1.DOCX]

Supplementary Material

Table S1: Description of play types

| **Play Types** | **Description** |
| --- | --- |
| Restorative play | talking about the play activity or chatting in a playful way, joking, reading a book, writing, listening to music |
| Physical play | climbing, running, throwing, lifting, jumping, spinning, swinging, skipping, using monkey bars wrestling, tussling, tumbling etc. |
| Exploratory play | exploration of an object or environment, such as searching for bugs or flowers, stirring water and dirt etc. |
| Imaginative play | pretending to be someone or something else, either from social interpersonal experiences or characters from fantasy world |
| Expressive play | expressing self through performance, artistic ways or language play, such as singing, dancing, drawing, making up rhymes etc. |
| Chasing/hiding games | rule based games involving chasing, catching, hiding, seeking, such as forms of tag or hide-and-seek |
| Ball games | rule based ball games, such as football, basketball, tennis etc. |
| Other | any other play activity or game |

Table S2: Frequencies of activity and play types by year group and gender for both schools

|  | **SCHOOL A** | | | |  | **SCHOOL B** | | | |
| --- | --- | --- | --- | --- | --- | --- | --- | --- | --- |
|  | Y3/4 Boys | Y5/6 Boys | Y3/4 Girls | Y5/6 Girls |  | Y3/4 Boys | Y5/6 Boys | Y3/4 Girls | Y5/6 Girls |
| **Activity Types** |  |  |  |  |  |  |  |  |  |
| Conversations | 5 | 28 | 17 | 69 |  | 46 | 74 | 76 | 142 |
| Play | 284 | 272 | 282 | 192 |  | 355 | 280 | 304 | 250 |
| Other | 18 | 26 | 30 | 22 |  | 25 | 25 | 35 | 28 |
|  |  |  |  |  |  |  |  |  |  |
| **Play Types** |  |  | | |  |  |  | | |
| Restorative play | 9 | 16 | 16 | 27 |  | 32 | 58 | 57 | 58 |
| Physical play | 24 | 12 | 79 | 50 |  | 44 | 44 | 70 | 53 |
| Exploratory play | 8 | 13 | 19 | 8 |  | 15 | 8 | 13 | 2 |
| Imaginative play | 26 | 20 | 42 | 13 |  | 57 | 26 | 67 | 38 |
| Expressive play | 2 | 1 | 18 | 7 |  | 6 | 6 | 28 | 20 |
| Chasing/hiding games | 36 | 48 | 45 | 47 |  | 88 | 41 | 48 | 44 |
| Ball games | 175 | 154 | 33 | 31 |  | 96 | 95 | 7 | 24 |
| Other | 4 | 8 | 30 | 9 |  | 17 | 2 | 14 | 11 |

Table S3: Chi-square test results with standardized residuals for both schools

|  | **SCHOOL A** | | | |  | **SCHOOL B** | | | |  |
| --- | --- | --- | --- | --- | --- | --- | --- | --- | --- | --- |
|  | Y3/4 Boys | Y5/6 Boys | Y3/4 Girls | Y5/6 Girls |  | Y3/4 Boys | Y5/6 Boys | Y3/4 Girls | Y5/6 Girls |  |
| **Activity Types** |  | *χ²*(6) = 105.95, p < .001 | | |  |  | *χ²*(6) = 75.62, p < .001 | | |  |
| Conversations | **-4.49** | -0.57 | **-2.58** | **8.07** |  | **-4.46** | -0.47 | -1.03 | **5.96** |  |
| Play | 1.88 | 0.14 | 0.59 | **-2.75** |  | **2.63** | 0.32 | 0.18 | **-3.12** |  |
| Other | -1.17 | 0.17 | 0.92 | 0.04 |  | -0.8 | -0.22 | 1.2 | -0.17 |  |
|  |  |  |  |  |  |  |  |  |  |  |
| **Play Types** |  | *χ²*(21) = 311.25, p < .001 | | |  |  | *χ²*(21) = 206.46, p < .001 | | |  |
| Restorative play | **-2.25** | -0.46 | -0.61 | **4.02** |  | **-3.73** | 1.4 | 0.63 | **2.27** |  |
| Physical play | **-3.19** | **-4.78** | **5.03** | **3.47** |  | **-2.39** | -0.81 | **2.19** | 1.3 |  |
| Exploratory play | -1.44 | 0.09 | 1.62 | -0.32 |  | 1.08 | -0.32 | 1.05 | **-2.12** |  |
| Imaginative play | -0.35 | -1.29 | **2.73** | -1.34 |  | 0.12 | **-2.75** | **2.73** | -0.24 |  |
| Expressive play | **-2.06** | **-2.35** | **3.73** | 0.78 |  | **-2.81** | **-2.16** | **3.23** | **2.08** |  |
| Chasing/hiding games | -1.8 | 0.22 | -0.46 | **2.48** |  | **2.71** | -1.53 | -1.13 | -0.36 |  |
| Ball games | **6.4** | **4.93** | **-7.19** | **-4.94** |  | **3.65** | **5.91** | **-6.6** | **-3.32** |  |
| Other | **-2.68** | -1.49 | **4.29** | -0.16 |  | 1.07 | **-2.6** | 0.82 | 0.57 |  |
| Note: Standardized residual values beyond ±1.96 are considered significant contributions at the 5% level and made bold. | | | | | | | | | |  |

Table S4: Full Negative Binomial Regression Results for the Effect of VMD on Child Group Counts by Gender and Year Group

|  |  |  |  |  |  |  |  |  |  |  |
| --- | --- | --- | --- | --- | --- | --- | --- | --- | --- | --- |
| **SCHOOL A** | | |  |  |  |  |  |  |  |  |
|  |  |  |  |  |  |  |  |  |  |  |
| gender | year | β (VMD) | SE | df | 95%CI lower | 95%CI upper | z.ratio | p.value | IRR | %chng |
| boy | y34 | -0.276 | 0.069 | Inf | -0.412 | -0.140 | -3.976 | 0.000 | 0.76 | -24 |
| girl | y34 | 0.398 | 0.068 | Inf | 0.264 | 0.532 | 5.824 | 0.000 | 1.49 | 49 |
| boy | y56 | -0.641 | 0.070 | Inf | -0.779 | -0.503 | -9.101 | 0.000 | 0.53 | -47 |
| girl | y56 | -0.240 | 0.074 | Inf | -0.385 | -0.094 | -3.232 | 0.001 | 0.79 | -21 |
|  |  |  |  |  |  |  |  |  |  |  |
| **SCHOOL B** | | |  |  |  |  |  |  |  |  |
|  |  |  |  |  |  |  |  |  |  |  |
| gender | year | β (VMD) | SE | df | 95%CI lower | 95%CI upper | z.ratio | p.value | IRR | %chng |
| boy | y34 | -0.454 | 0.038 | Inf | -0.529 | -0.379 | -11.795 | 0.000 | 0.64 | -36 |
| girl | y34 | -0.351 | 0.043 | Inf | -0.434 | -0.268 | -8.245 | 0.000 | 0.70 | -30 |
| boy | y56 | -0.717 | 0.044 | Inf | -0.803 | -0.631 | -16.305 | 0.000 | 0.49 | -51 |
| girl | y56 | -0.132 | 0.042 | Inf | -0.214 | -0.049 | -3.129 | 0.002 | 0.88 | -12 |

Table S5: Full Negative Binomial Regression Results for the Effect of VMD on Child Group Counts by Gender and Year Group (Ball-Game Observations Excluded)

|  |  |  |  |  |  |  |  |  |  |  |
| --- | --- | --- | --- | --- | --- | --- | --- | --- | --- | --- |
| **SCHOOL A** | | |  |  |  |  |  |  |  |  |
|  |  |  |  |  |  |  |  |  |  |  |
| gender | year | β (VMD) | SE | df | 95%CI lower | 95%CI upper | z.ratio | p.value | IRR | %chng |
| girl | y34 | -0.015 | 0.072 | Inf | -0.156 | 0.125 | -0.213 | 0.832 | 0.98 | -2 |
| boy | y34 | 1.025 | 0.079 | Inf | 0.870 | 1.179 | 12.982 | 0.000 | 2.79 | 179 |
| girl | y56 | -0.834 | 0.079 | Inf | -0.988 | -0.679 | -10.600 | 0.000 | 0.43 | -57 |
| boy | y56 | 0.530 | 0.075 | Inf | 0.382 | 0.678 | 7.029 | 0.000 | 1.70 | 70 |
|  |  |  |  |  |  |  |  |  |  |  |
| **SCHOOL B** | | |  |  |  |  |  |  |  |  |
|  |  |  |  |  |  |  |  |  |  |  |
| gender | year | β (VMD) | SE | df | 95%CI lower | 95%CI upper | z.ratio | p.value | IRR | %chng |
| girl | y34 | -0.389 | 0.045 | Inf | -0.476 | -0.302 | -8.734 | 0.000 | 0.68 | -32 |
| boy | y34 | -0.266 | 0.042 | Inf | -0.348 | -0.184 | -6.357 | 0.000 | 0.77 | -23 |
| girl | y56 | -0.074 | 0.045 | Inf | -0.161 | 0.014 | -1.647 | 0.100 | 0.93 | -7 |
| boy | y56 | -0.329 | 0.048 | Inf | -0.423 | -0.235 | -6.848 | 0.000 | 0.72 | -28 |
